# The Role of the Piezo1 Mechanosensitive Channel in Heart Failure

**DOI:** 10.3390/cimb45070369

**Published:** 2023-07-13

**Authors:** Weihua Yuan, Xicheng Zhang, Xiangming Fan

**Affiliations:** 1 National Clinical Research Center for Child Health, Children’s Hospital, Zhejiang University School of Medicine, 3333 Binsheng Rd, Hangzhou 310052, China; ywh038943@163.com; 2 National Clinical Research Center for Child Health, Department of Cardiac Surgery, Children’s Hospital, Zhejiang University School of Medicine, 3333 Binsheng Rd, Hangzhou 310052, China; xicheng_zhang@zju.edu.cn

**Keywords:** Piezo1 channels, heart failure, mechanotransduction, Ca^2+^

## Abstract

Mechanotransduction (MT) is inseparable from the pathobiology of heart failure (HF). However, the effects of mechanical forces on HF remain unclear. This review briefly describes how Piezo1 functions in HF-affected cells, including endothelial cells (ECs), cardiac fibroblasts (CFs), cardiomyocytes (CMs), and immune cells. Piezo1 is a mechanosensitive ion channel that has been extensively studied in recent years. Piezo1 responds to different mechanical forces and converts them into intracellular signals. The pathways that modulate the Piezo1 switch have also been briefly described. Experimental drugs that specifically activate Piezo1-like proteins, such as Yoda1, Jedi1, and Jedi2, are available for clinical studies to treat Piezo1-related diseases. The only mechanosensitive ion-channel-specific inhibitor available is GsMTx4, which can turn off Piezo1 by modulating the local membrane tension. Ultrasound waves can modulate Piezo1 switching in vitro with the assistance of microbubbles. This review provides new possible targets for heart failure therapy by exploring the cellular functions of Piezo1 that are involved in the progression of the disease. Modulation of Piezo1 activity may, therefore, effectively delay the progression of heart failure.

## 1. Introduction

The heart is a critical organ that transports nutrients and oxygen throughout the body. Heart failure (HF) is a major cardiovascular disease and one of the leading causes of morbidity and mortality worldwide. HF is a syndrome characterized by diastolic or systolic myocardial and circulatory dysfunction, resulting in cardiac output that is insufficient to maintain tissue metabolism [1]. Almost all cardiovascular diseases eventually cause heart failure. Various pathophysiological changes, including myocardial ischemia and metabolic dysregulation, genetic alterations of cardiac protein function and response infarction, pressure, or volume overload linked to viral infection, can trigger HF [2]. The annual mortality rate for HF patients worldwide is over 10% because of sudden cardiac death and multi-organ dysfunction [3].

HF can be classified using various approaches. It is divided into left heart failure, right heart failure, and total heart failure, according to the leading site of heart failure and clinical manifestations. Additionally, HF is classified as acute or chronic based on the time taken for symptoms to appear. Furthermore, there are two forms of HF based on changes in ejection fraction values. These include (i) HF with preserved ejection fraction (HFpEF) and (ii) HF with reduced ejection fraction (HFrEF). Patients with HFpEF have an aberrant ejection fraction of >50% or <40%. The prevalence of HFpEF has increased in recent years. Moreover, the lack of effective medicines that can treat HFpEF has resulted in poor prognosis. A considerable loss of cardiomyocytes (CMs) during myocardial infarction (MI) leads to reduced ejection fraction, resulting in HFrEF.

Regardless of the underlying cause, HF is associated with the local and systemic activation of the inflammatory signaling cascade [4]. Inflammation is the primary cause of HF in patients with myocarditis and inflammatory cardiomyopathies [5,6]. In most other circumstances, activation of the inflammatory program is a reparative or protective response to other primary harmful events. In HF, the inflammatory response is characterized by the production and activation of various pleiotropic cytokines and chemokines that influence the phenotype and function of CMs. Excessive, uncontrolled, or dysregulated inflammation, regardless of the underlying cause, may aggravate myocardial damage and contribute to HF progression.

The heart is intrinsically able to adapt its cardiac output to changes in mechanical load. Cells can detect changes in mechanical stress in the microenvironment and adjust to new mechanical requirements. Molecular mechanotransduction (MT) mechanisms and their effects on cardiac health and disease remain elusive. Various mechanical factors can induce cardiac injury resulting in HF, but cells sense harmful stimuli through MT [7]. Mechanical forces can generally be categorized based on the type of force, such as blood flow shear, tensile stress, and stiffness. Different mechanical pressures can induce different stages of inflammation. For instance, abnormalities in blood flow shear can harm the vascular endothelial cells, eventually resulting in atherosclerosis [8]. Inflammation caused by myocardial overload leads to long-term heart damage, such as myocardial fibrosis and hypertrophy [9]. All the cells involved in HF exhibit sensitivity to mechanical forces, such as the deformation of cardiac fibroblasts (CFs) under mechanical stimulation and the “extrusion” of immune cells from blood vessels and migration to the site of inflammation [10]. Mechanosensitive ion channels are the leading proponents of mechanoelectrical feedback because they rapidly respond to mechanical stimulation and can directly affect cardiac electrical activity [11]. Piezo1 is a recently discovered mechanosensitive ion channel protein that is an efficient cellular mechanosensor [12]. It converts mechanical impulses into electrical or chemical signals using Piezo1 [13]. Piezo1 responds to mechanical stimuli by generating depolarizing ionic currents and cytoplasmic Ca^2+^ elevations, which are both profound modulators of cell function [14]. Under mechanical stress, Piezo1 regulates various processes including protein synthesis, secretion, migration, proliferation, and apoptosis [15,16,17,18]. It plays important roles in cardiovascular physiology and disease as it is involved in cardiovascular maturation and cardiovascular force sensing [19,20]. In addition, Piezo1 plays a crucial role in acute blood pressure regulation [21]. It is expressed in CMs and involved in sensing the dramatic mechanical changes produced by cardiac systole and diastole, thereby regulating cardiac homeostasis. Piezo1 serves as a key cardiac mechanotransducer that initiates mechano–chemo transduction and consequently maintains normal heart function [22]. It is also required for outflow tract and aortic valve development [23]. Piezo1 expression is upregulated in patients with heart disease [24]. Furthermore, Piezo1 regulates heart homeostasis by converting mechanical forces into electrical signals. In response to various acute or prolonged harmful mechanical signals, Piezo1 plays an essential role in HF by converting mechanical stimuli into intracellular pro-inflammatory signals. This enables the heart to sense changes in mechanical force and respond accordingly. The exact mechanism by which Piezo1 functions as a mechanical force sensor in the heart and its role in the progression of heart failure remains to be explored.

In this review, we summarize the roles of Piezo1 in different types of the heart cells, particularly in the signaling pathways involved in Piezo1 regulation in HF.

## 2. Piezo1

### 2.1. Piezo1 Structure

**Figure 1:**
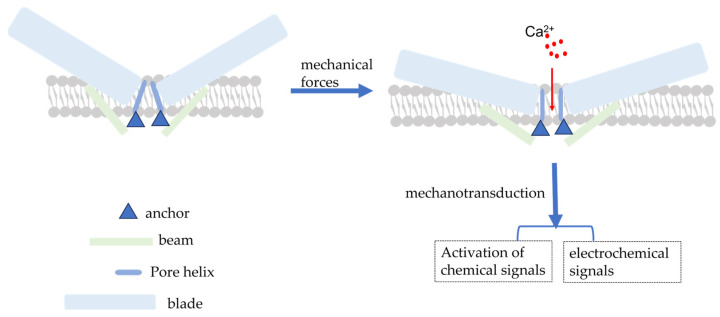
Schematic diagram of the simple structure of piezo1 and piezo1 activated by mechanical force. Mechanical forces applied to the cell membrane cause the opening of Piezo1 channels, resulting in the influx of extracellular Ca^2+^ and the conversion of mechanical signals into electrochemical and chemical signals.

The *PIEZO1* gene, located on human chromosome 16q24.3, has 51 exons, encodes 2520 amino acid residues, and has a molecular weight of approximately 320 kDa [15,25]. Coste (2010) was the first to investigate for Piezo1 expression using electrophysiological techniques and mechanical force stimulation (a black needle penetrating the cell membrane). Ge et al. (2015) published the cryo-electron microscopic structure of recombinant full-length mouse Piezo1 with a resolution of 4.8 Å [26]. Moreover, Saotome et al. and Zhao et al. presented mouse Piezo1 protein structures with resolutions of 3.8 Å and 3.97 Å, respectively [27,28]. Briefly, the Piezo1 channel is a trimeric three-leaf propeller-like structure with a central pore portion (*C*-terminus) responsible for ion permeation, three peripheral paddle portions (*N*-terminus) responsible for mechanical force perception, and a transduction portion consisting of a crossbeam and an anchor. This structure is required for MT [28,29] (Figure 1).

### 2.2. Piezo1 Activation Mechanism

The Piezo1 channel can be activated by a series of mechanical forces, such as membrane indentation, membrane stretching, shear stress, and hydrostatic pressure, which cause changes in membrane tension [30]. When cells are subjected to mechanical force, Piezo1 channels are distributed across the cell membrane and the lipid interactions surrounding the channels form a dome-like structure, making them more sensitive to mechanical stimulation [31]. Simultaneously, Na^+^, Ca^2+^, and other ions cause membrane potential depolarization to effectively convert the mechanical force process into electrochemical signals [32,33] (Figure 1). However, the exact mechanism of Piezo1 activation by mechanical force remains to be elucidated. There has been some progress but it is still a hypothesis; the following mechanisms still exist. Firstly, membrane tension is heterogeneous across the membrane [34] and cellular traction likely produces a local increase in membrane tension that can cause reversible deformation of the Piezo1 protein from a curved to a spreading phenotype, causing expansion of the planar membrane area and driving the opening of Piezo1 channels [34,35]. Simon Scheuring et al. used cryo-electron and high-speed atomic force microscopy to demonstrate that Piezo1 can deform into a planar structure. This deformation could explain how lateral membrane tension can gate the Piezo1 channel in response to mechanical perturbations [36,37]. Based on the aforementioned energetic discussion, force-induced membrane tension opens the channel, allowing the permeation of cations [38]. Secondly, cytoskeletal proteins can alter the membrane tension and direct physical interactions between Piezo1 channels and the cytoskeleton can lead to Piezo1 activation [31,39,40]. In human neural stem cells, the actin cytoskeleton generates sufficient force to open Piezo1 through actin II phosphorylation [41]. The cortical cytoskeleton inhibits membrane stretching under mechanical manipulation, thereby providing mechanical protection against Piezo1 [38]. Thirdly, similar to integrins, Piezo1 activation can be altered by stimuli from within or outside the cell [42]. Protein–protein interactions can also activate Piezo1. For example, stomatin-like protein-3 controls membrane mechanics by binding to cholesterol, facilitating force transmission, and modulating Piezo channel sensitivity [43]. In addition, Xiao et al. identified a new interacting protein of Piezo1, sarcoplasmic/endoplasmic reticulum Ca^2+^ ATPase (SERCA2). The site in Piezo1 responsible for the interaction with SERCA2 is a linkage region between the pore region and the mechanically gated structural domain, through which SERCA2 can inhibit the activity of Piezo channels [44].

Both mechanical forces and interacting proteins ultimately activate Piezo1, leading to ion influx. Although Piezo1 channels are non-selective cation channels, they are more permeable to Ca^2+^ than to other positive ions [45,46]. Furthermore, experiments have shown that signal transduction through the Piezo1 channel occurs via Ca^2+^, which acts as a secondary messenger in the signal transduction pathway, regulating cell activation and function [47,48,49].

## 3. The Necessity for Piezo1 in HF

When destructive mechanical forces are applied to cells, in addition to the direct mechanical damage to the structural cells of the heart, indirect damage is induced by the secretion of pro-inflammatory factors. Inflammation and fibrosis are fundamentally intertwined mechanisms in HF [50]. Piezo1 acts as a novel mechanosensor in the heart and regulates cardiac injury repair through its expression in various cells during HF. The cells involved in the repair process express Piezo1 and are mechanically stimulated to varying degrees. These cells include endothelial cells (ECs), CFs, and CMs, with CFs exhibiting higher expression levels than CMs [51,52]. Piezo1 is expressed in many immune cells, including macrophages, dendritic cells (DCs), and T cells. The pro-inflammatory cytokines tumor necrosis factor α (TNF-α), interleukin (IL)-1, and IL-6 secreted by immune cells are widely associated with the pathogenesis of HF. Chemokines (e.g., CCL2) are also upregulated in HF and may stimulate the recruitment of pro-inflammatory leukocytes, promoting myocardial injury, fibrotic remodeling, and dysfunction [53]. CCL2 chemokines exert myocardial fibrosis through the recruitment and activation of CCR2-expressing monocytes and macrophages [54]. CCL17 levels increase with age and correlate with cardiac dysfunction. Animal experiments further revealed that Ccll7-KO significantly retarded aging and repressed angiotensin II (Ang II)-induced cardiac hypertrophy and fibrosis, which are accompanied by the plasticity and differentiation of T-cell subsets [55]. The lymphatic vessels play a crucial pathophysiological role in promoting fluid and immune cell tissue clearance. Conversely, immune cells may affect lymphatic function and remodeling. Cardiac lymphatic vessels are vital in maintaining cardiac homeostasis under physiological and pathological conditions. In major cardiovascular diseases, including atherosclerosis, myocardial infarction (MI), and heart failure, cardiac lymphatics regulate the transportation of extravasated proteins and lipids, inflammatory and immune responses, and fluid balance [56]. Recently, Houssari et al. determined the effect of cardiac-infiltrating T cells on cardiac lymphatic remodeling and proposed cardiac lymphangiogenesis as a therapeutic target for preventing HF after MI [57].

### 3.1. Cardiac Structural Cells

#### 3.1.1. CFs

In healthy hearts with normal function, CFs are mostly quiescent and embedded in a structurally stable extracellular matrix (ECM) [58]. However, in HF, CFs extend in all directions because of cardiac injury as they respond to stressful stimuli and adapt to changing environments. During cardiac injury repair, fibroblasts transdifferentiate into myofibroblasts, resulting in increased proliferation and migration capacities, contractile properties, and enhanced secretion of ECM proteins, growth factors, and cytokines, which regulate the local cellular environment through paracrine signaling [59]. In vitro, CFs respond to multiple mechanical forces such as cell membrane deformation by stretching, cyclic stretching, or compression [60,61,62]. Cell membrane deformation also occurs by promoting cellular ion influx, particularly calcium influx [63]. Piezo1 mediates stretch-induced expression of brain natriuretic peptides in adult rat heart fibroblasts [64]. Blythe et al. investigated Piezo1 expression and functional activity in human and mouse CFs and found that Piezo1 activation was coupled with the activation of the p38α MAPK pathway and IL-6 secretion. In addition, IL-1 secretion in CFs cultured on softer collagen-coated substrates was reduced by Piezo1 knockdown, suggesting that culture conditions (substrate composition and stiffness) may alter Piezo1 signaling in vitro [51]. Increased atrial fibroblast load or cardiac stretch can activate Piezo1 to increase Ca^2+^ influx and promote inflammation, fibroblast proliferation, and myocardial fibrosis [65].

**Figure 2:**
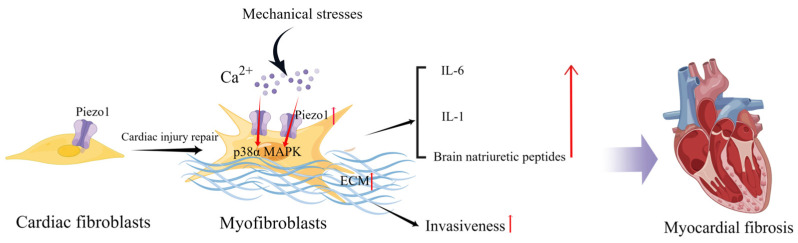
Schematic diagram of the mechanism of action of Piezo1 in cardiac fibroblasts. Mechanical stress induces Piezo1 opening in fibroblasts and promotes IL-6 expression via the p38α MAPK signaling pathway. Mechanical stretch induces fibroblasts to secrete natriuretic peptides and ECM, and the increase in matrix stiffness may further upregulate Piezo1 expression and promote fibrosis progression (created with https://www.figdraw.com/static/index.html accessed on 5 June 2023).

Interestingly, CFs isolated from patients with atrial fibrillation expressed higher levels of Piezo1 and increased Piezo1 activity compared with CFs from patients with sinus rhythm, suggesting that Piezo1 also plays a potentially important role in atrial fibrillation and remodeling [66]. Piezo1 increases atrial fibroblast stiffness by increasing the number and thickness of the F-actin network cytoskeletal components in response to increased ECM stiffness [67]. Additionally, Piezo1 overexpressing cells can increase the stiffness of neighboring non-transfected cells via a paracrine IL-6 signaling [67]. Piezo1 activity stimulates ECM and cytokine production, thereby promoting the phenoconversion of adjacent fibroblasts into new myofibroblasts and enhancing invasiveness. Furthermore, global functionally acquired mutations in Piezo1 in mice increase calcium flow, activate downstream p38 signaling, and enhance IL-6 secretion in cardiac fibroblasts [68] (Figure 2). Activation of Piezo1 may be a key driver of cardiac remodeling and myofibroblast persistence [69]. Therefore, inhibition of Piezo1 activity may also assist in cardiac fibrotic remodeling. Modulation of Piezo1 expression and activation in fibroblasts can influence the progression of myocardial fibrosis, including the degree of fibrosis and inflammatory processes and thus the outcome of HF.

#### 3.1.2. CMs

Expression of Piezo1 is up-regulated during the heart failure process [70]. Xiang et al. found that Piezo1, as a preference for Ca^2+^, is remarkably up-regulated under pressure overload and is enriched near the T-tubule and the intercalated disc of the cardiomyocyte. Additionally, using cardiac conditional Piezo1 knockout mice undergoing transverse aortic constriction, they demonstrated that Piezo1 was required for the development of cardiac hypertrophy and subsequent adverse remodeling [71]. Indeed, an autonomic upregulation of Piezo1 in cardiomyocytes contributes to cardiomyopathy in both cellular and animal models and human patients that might have altered mechanical stress conditions [22]. Jiang et al. found that the Piezo1 channel mediates the stretch-induced Ca^2+^ response of CMs, which in turn produces ROS. Their study revealed moderate enlargement of the left ventricle in the hearts of piezo1KO mice, markedly enhanced interstitial fibrosis in heart slices, and reduced the ejection fraction. Doxorubicin-induced dilated cardiomyopathy increases Piezo1 expression in CMs, and clinical data have shown increased Piezo1 expression in cardiac biopsies from patients with hypertrophic cardiomyopathy [22]. Rolland et al. demonstrated that the mechanosensitive ion channel Piezo1 was expressed in zebrafish CMs. Furthermore, chemically prolonging Piezo1 activation in zebrafish resulted in cardiac arrhythmias [72].

**Figure 3:**
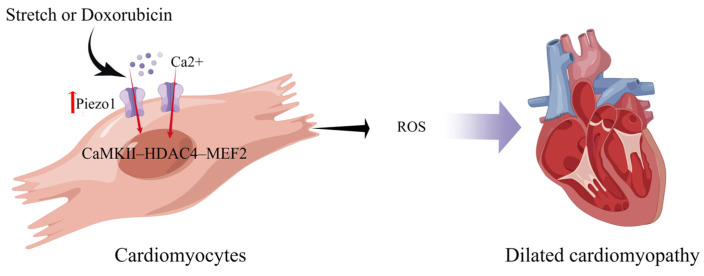
Schematic diagram of the mechanism of action of Piezo1 in cardiomyocytes. Piezo1 helps cardiomyocytes transduce mechanical stretch into intracellular Ca^2+^ signaling leading to ROS production. Mechanical overload results in increased Piezo1 expression in cardiomyocytes via the CaMKII-HDAC4–MEF2 pathway, ultimately leading to dilated cardiomyopathy (created with https://www.figdraw.com/static/index.html accessed on 5 June 2023).

Piezo1, which is expressed in CMs, is a mechanosensor that initiates the CaMKII–HDAC4–MEF2 hypertrophic signaling cascade to induce left ventricular hypertrophy in response to pressure overload [71] (Figure 3). CM-specific deletion of Piezo1 in adult mice prevents CaMKII activation and inhibits hypertrophic responses [73]. piezo1 has no protective effect for cardiomyocytes in healthy hearts; however, pathological states or gene overexpression can lead to severe myocardial hypertrophy and arrhythmias [22,71]. piezo1 is expressed in cardiac myocytes appropriately, but too much can damage the heart.

#### 3.1.3. ECs

**Figure 4:**
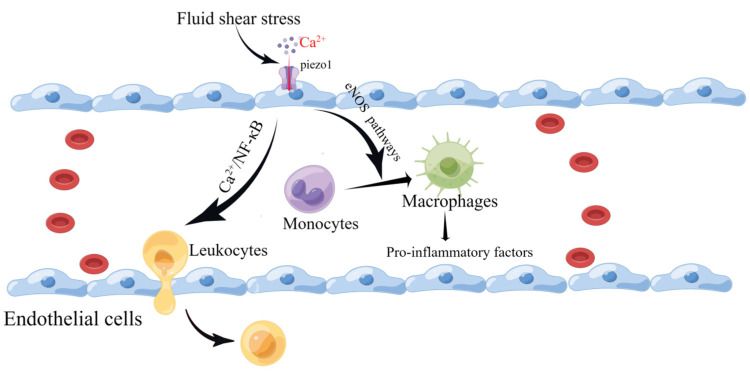
Schematic diagram of the role of Piezo1 in vascular endothelial cells. Piezo1 senses fluid shear, leading to Ca^2+^ influx that activates the eNOS signaling pathway and contributing to the differentiation of monocytes into macrophages. In addition, it induces leukocyte entry and exit by activating the NF-κB pathway to open the endothelial barrier (created with https://www.figdraw.com/static/index.html accessed on 5 June 2023).

Piezo1 mRNA has been detected in the mouse aorta and various human endothelial cells. Vascular endothelial cells are affected by the intra- and extra-luminal environment of the vascular lumen. They are endowed with different mechanical forces, such as shear stress caused by blood flow and tension, that are sensed and transmitted by Piezo1. This leads to altered cellular behavior, over-proliferation, migration, and aggregation, forming a primitive vascular system and leading to further pathological vascular remodeling [74,75,76,77]. Wang et al. reported that the mechanosensitive cation channel Piezo1 plays a crucial role in the transendothelial migration of leukocytes in vitro and in vivo by activating Piezo1 through low levels of fluid shear stress that mediate Ca^2+^ as well as the local opening of the endothelial barrier [78]. Changes in plasma membrane tension are restricted to the subcellular structural domains of endothelial cells, as local increases in membrane tension result only in local activation of the mechanosensitive ion channel Piezo1 [79]. Atherosclerosis is a multifactorial inflammatory disease initiated by vascular endothelial cell damage. Following endothelial injury, cholesterol and lipids in the blood are deposited under the endothelium, attracting monocytes to accumulate and differentiate into macrophages [80]. Piezo1 inhibits the vascular-remodeling-related signaling pathways P2Y2 and Gq/G11 under high-shear-stress laminar flow but activates downstream eNOS signaling pathways, resulting in protective effects against atherosclerotic disease [81,82,83,84]. Han et al. found that silencing Piezo1 expression in human umbilical vein endothelial cells (HUVEC) and mouse liver endothelial cells (MLEC) protected against atherosclerosis [85]. Vascular endothelial cells continue to be subjected to shear forces from blood flow, and Piezo1 activation leads to macrophage activation [86,87] and the phagocytosis of lipids into foam cells, which secrete pro-inflammatory factors [88]. The widening of blood flow channels to reduce high shear stress also reduces the pro-inflammatory action of monocytes [80]. In addition, Piezo1 channels on vascular endothelial cells are activated by turbulence and induce inflammatory signaling through integrin-associated PECAM-1/VE-calmodulin/VEGFR2 and PI3-kinase-dependent activation, leading to FAK-dependent NF-κB activation and reduced eNOS excitation by activating phosphodiesterase 4D to promote cAMP degradation [83]. NF-κB activation leads to the production of leukocyte adhesion molecules (including vascular cell adhesion molecule 1 and intercellular adhesion molecule-1) and chemokines (including CCL2) that promote AS progression [89,90,91] (Figure 4). NF-κB also promotes NOD-like receptor thermal protein domain associated protein 3 (NLRP3) assembly, cystatin-1 triggering, and IL-1β production [92].

### 3.2. Immune Cells

**Figure 5:**
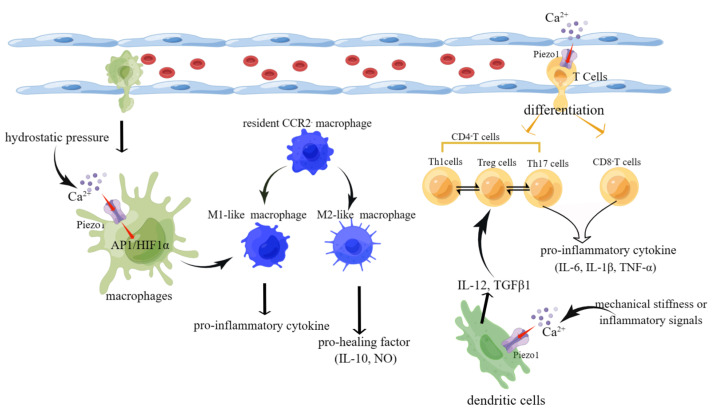
Schematic diagram of the role of Piezo1 in immune cells. (1) Activation of piezo1 upon stimulation by hydrostatic pressure leads to Ca^2+^ influx. In turn, the increase in Ca^2+^ activates the AP1/HIF1ɑ signaling pathway, inducing macrophage differentiation into M1-like macrophages that secrete pro-inflammatory cytokines. (2) Mechanical stiffness or inflammatory signals activate piezo1 on DCs, leading to Ca^2+^ influx and secretion of polarizing factors IL-12 and TGF-β and ultimately affecting the mutual differentiation of Th1 and Treg cells. (3) Activation of piezo1 on T cells affects their differentiation into CD^4+^/CD^8+^ T cells and Th17/Treg cell reciprocally (created with https://www.figdraw.com/static/index.html accessed on 5 June 2023).

The heart failure process involves not only the heart’s structural cells but also various immune cells. Most immune cells, such as M1-like macrophages, CD8+ T cells, and Th17 cells, secrete mainly pro-inflammatory factors to promote the progression of heart failure. However, M2-like macrophages and Treg cells secrete pro-healing factors slowing heart failure (Figure 5).

#### 3.2.1. Macrophages

Macrophages are multifunctional cells of the innate immune system that play key roles in homeostasis, pathogen defense, and response to injury in vivo. This diversity of macrophage function is because of their ability to respond dynamically to microenvironmental changes and polarize toward multiple functional phenotypes [93,94,95]. Macrophages are divided into M1 (the classical pro-inflammatory phenotype) and M2 (the non-classical anti-inflammatory phenotype) types [96]. Mechanical loading can affect macrophage function, leading to excessive and uncontrollable inflammation and systemic damage, including cardiovascular disease and knee osteoarthritis. Mechanical stretch modulates macrophage polarization and enhances the inflammatory response in vitro [97].

Bajpai et al. demonstrated that the human myocardium contains different subpopulations of CCR2^−^ and CCR2^+^ macrophages. CCR2^−^ macrophages are tissue-resident populations replenished by local proliferation, whereas CCR2^+^ macrophages are maintained by monocyte recruitment and proliferation. In addition, CCR2^−^ and CCR2^+^ macrophages have distinct functional properties similar to those of CCR2^−^ and CCR2^+^ macrophages in the mouse heart [98]. CCR2^−^ macrophages are involved in various forms of tissue remodeling, such as coronary artery development, vasodilation, and cardiac tissue repair [99,100]. For example, after neonatal CM injury, CCR2^−^ macrophages coordinate cardiac tissue regeneration and functional recovery of the heart through the coronary vascular system, CM expansion, proliferation, and physiological CM hypertrophy. Following myocardial ischemia and reperfusion injury, CCR2^+^ macrophages are activated in a TLR9-dependent manner and coordinate neutrophil extravasation into the injured myocardium to produce the neutrophil chemokines CXCL2 and CXCL5 [101].

Piezo1 is subjected to different forms of mechanical stimulation that affect macrophage phenotype transformation, mainly toward an M1-like pro-inflammatory phenotype or CCR2^+^ macrophages. Myeloid-derived suppressor cells are cardioprotective in HF because of their anti-hypertrophic effects on CMs and anti-inflammatory effects via IL-10 and NO [102]. Bone-marrow-derived macrophages (BMDMs) highly express Piezo1 channel proteins. Piezo1 senses periodic hydrostatic pressure (CHP) changes, leading to intracellular AP-1 activation that promotes EDN1 expression and stabilizes hypoxia-inducible factor-1α (HIF1α) and, ultimately, pro-inflammatory cytokine expression [103]. In macrophages stimulated by bacterial infection or lipopolysaccharide (LPS), Toll-like receptor 4 and Piezo1 form a complex that leads to the activation of Piezo1. Ca^2+^ influx activates CaMKII, phosphorylates and activates Mst1/2, and finally activates the Rac signaling pathway to promote phagocytosis and bacterial clearance by macrophages [104].

Substrates with different stiffnesses also affect macrophage polarization [105]. Atcha et al. found increased Piezo1 expression in BMDMs cultured on stiffer substrates, which increased Ca^2+^ influx. They promoted “M1-like” pro-inflammatory cytokine production and inhibited “M2-like” pro-healing factor secretion, ultimately enhancing macrophage clearance. Thus, Piezo1 can act as a mechanical stiffness sensor in macrophages. In myocardial fibrosis, matrix stiffening may promote increased Piezo1 expression in macrophages, thereby exacerbating the inflammatory response. In addition, this study demonstrated a positive feedback loop between Piezo1 and the cytoskeleton of macrophages, with inhibition of actin polymerization and reduction of Ca^2+^ influx through Piezo1 [86].

Mechanical forces, such as cyclic or static stretching, cause morphological differences in macrophages and affect their proliferation. Static and cyclic stretching increase the expression of integrin CD11b (ɑM integrin), which inhibits the expression of Piezo1 and ultimately causes a stretch-mediated inflammatory response [106]. During myocardial injury, macrophages squeeze and stretch from the endothelial cell layer into the damaged myocardial layer, activating Piezo1 on the macrophages to promote cardiac injury repair and indirectly accelerate myocardial fibrosis. Mechanical force upregulates Piezo1 expression in periodontal tissues and cultured BMDMs. Piezo1 enhances Rb1 phosphorylation by activating downstream cyclin D1, thereby promoting macrophage proliferation [107].

Upon bacterial infection or activation by LPS, macrophages undergo significant changes in glucose metabolism, including aerobic glycolysis, the pentose phosphate pathway, and the tricarboxylic acid cycle [108]. Leng et al. found that Piezo1 knockout macrophages reduced aerobic glycolysis at rest and in response to LPS stimulation. After treatment with Piezo1 agonist Yoda1 or CHP, Piezo1 channels open, leading to Ca^2+^ influx that induces the CaMKII-HIF1ɑ pathway to promote aerobic glycolysis and LPS-induced macrophage immune responses [109]. In addition to directly affecting glucose metabolism in macrophages, Piezo1 indirectly affects iron metabolism in the blood. Macrophages in the spleen and liver remove aged red blood cells through phagocytosis and degrade hemoglobin, eventually releasing iron into the blood [110]. Patapoutian et al. found that constitutive or macrophage expression of a gain-of-function Piezo1 allele in mice disrupted hepcidin levels and caused iron overload. Furthermore, Piezo1 is a crucial regulator of macrophage phagocytic activity and subsequent erythrocyte turnover [111].

#### 3.2.2. DCs

DCs are considered sentinels of the human immune system. Intrinsic cellular mechanisms and complex external environmental signals give DCs the unique ability to induce protective immunity or self-tolerance [112]. Dilated cardiomyopathy is the most common cause of HF in young patients and often results from autoimmunity triggered by viral or bacterial infections. DCs play an important role in inducing autoimmune myocarditis [113]. Following ischemic injury to the myocardium, DCs respond to myocyte necrosis, present cardiac antigens to T cells, and may trigger a sustained autoimmune response against the heart. The activation of cytotoxic CD8^+^ T cells by cross-initiating DCs worsens post-ischemic inflammatory injury and decreases cardiac function [114]. Immature DCs have a strong migratory capacity. Mature DCs can effectively activate naïve T cells and are central to the initiation, regulation, and maintenance of the immune response. As early as 2009, Craig et al. demonstrated that mechanical stress causes DCs to mature spontaneously [115]. Substrate stiffness and mechanical force control human DC agglutinin expression [116]. Substrate stiffness contributes to the differentiation of bone marrow-derived DCs in vitro. Their proliferation, activation, cytokine secretion capacity, and activation and flux of glucose metabolic pathways increased when grown at a higher stiffness [117]. However, the effects of tension on DCs may be transient and reversible, allowing the modulation of DC activation and thus enhancing the immunostimulatory potential of DCs. Tension allows DCs to stimulate an adaptive immune response, and DCs play an essential role in antitumor immunity by inducing T-cell differentiation [118]. Recently, Wang et al. found that Piezo1 stimulated by DCs through mechanical stiffness or inflammatory signals directed the mutual differentiation of Th1 and regulatory T (Treg) cells. The specific mechanism is the Ca^2+^-dependent SIRT1 HIF1α and calcium–calcineurin–NFAT signaling pathways, which use DC-derived IL-12 and TGFβ1 to coordinate the mutual differentiation of Th1 cells and Tregs [119]. Tregs protect against adverse ventricular remodeling and contribute to improved cardiac function after MI by inhibiting inflammation and directly protecting CMs [120]. This regulates the activation or switching of Piezo1 on DCs to regulate the activity of DCs and differentiation of Tregs, thereby affecting the process of HF.

#### 3.2.3. T Cells

Myocardial inflammation can lead to fatal acute or chronic HF. T lymphocytes of different etiologies of HF have been reported in inflamed hearts, and different T-cell subsets play different roles in the heart depending on the inflammatory triggering event [121]. T cells are significant contributors to nonischemic HF, and a reduction in T-cell infiltration has been identified as a novel therapeutic target for HF [122]. T-cell depletion confers late cardioprotection; however, heart pressure overload induced by cardioprotective hypertrophy occurs in the early stages of HF. Single-cell RNA sequencing analysis revealed that CD8^+^ T-cell depletion induces cardioprotective hypertrophy characterized by the expression of mitochondrial and growth factor receptor genes. CD8^+^ T cells regulate the conversion of cardiac-resident macrophages into cardioprotective macrophages expressing growth factor genes such as *AREG*, *OSM* and *IGF1* [122]. The depletion of CD8^+^ T cells decreases ischemic cardiomyocyte apoptosis, blocks inflammatory responses, limits myocardial damage, and improves cardiac function [123]. CD4^+^ T cells play a vital role in the progression of compensated cardiac hypertrophy to HF [123,124]. CD4^+^ T-lymphocytes are comprehensively expanded and activated in chronic ischemic HF, whereas differentiated Th2 and Th17 predominate in HF [125]. The Th17/Treg balance is involved in the pathological process of heart failure; maintaining the relative balance between them can delay the onset and progression of heart failure. Th17 cells and Treg cells, both derived from naive Th cells, have opposite roles. Th17 cells amplify the inflammatory response by promoting the secretion of IL-6, IL-1β, and TNF-α, whereas Treg cells secrete IL-10 to suppress the inflammatory response [126,127]. Th17 cells upregulate LOX expression by activating the IL-17/ERK1/2-AP-1 pathway, whereas Tregs downregulate LOX expression by activating the IL-10/JAK1-STAT3 pathway. A Th17/Treg imbalance affects the progression of myocardial fibrosis by deregulating LOX expression [128].

T lymphocytes often encounter complex mechanical signals during immune responses. Substrate stiffness and mechanical force can help initiate T-cell activation [129,130]. Meng et al. identified YAP as a critical inducer of T-cell inflammation on rigid substrates; however, it was able to reduce inflammation on soft substrates [131]. Piezo1 can be activated in response to membrane stretching, further enhancing the T-cell immune response to malarial infection [132] and contributing to lymphatic vessel formation [133,134]. Xie et al. verified that external mechanical forces can optimize T-cell activation. They used nanomotors to deliver a mechanical force to achieve localized immune cell stimulation, avoiding widespread T-cell activation and systemic immune storms [135]. It is through Piezo1 that the mechanical force is delivered to the immune synapse between the T-cell receptor and the central histocompatibility molecule, activating downstream signaling pathways and ultimately activating T cells [136,137]. However, in pathological conditions, such as multiple sclerosis and autoimmune encephalomyelitis, the presence of Piezo1 enhances the T-cell immune response and exacerbates symptoms. Furthermore, these data suggest that Piezo1 selectively inhibits Tregs without affecting T-cell activation or effector T-cell function [138]. Tregs benefit the heart by suppressing excessive inflammatory responses and promoting stable scar formation during the early stages of cardiac injury [120,139]. However, in chronic HF, Tregs undergo phenotypic and functional changes. They are converted into antiangiogenic and profibrotic cell types [140]. Tregs play a similar role in myocardial ischemia/reperfusion injury in mice [141]. C-C motif chemokine 17 exacerbates myocardial injury by inhibiting Treg recruitment [142]. Treg cells contribute to the resolution of inflammation and thus help in slowing down the progression of heart failure [139]. Regulating Piezo1 in T cells affects their differentiation and proliferation. Downregulation of Piezo1 expression significantly reduces the number of pro-inflammatory cells (CD8^+^ T cells and Th17 cells) and increases the number of Treg cells, which secrete anti-inflammatory cytokines that eventually retard the progression of heart failure.

## 4. Potential Applications of Piezo1 Research

### 4.1. Drug Development

Given the critical function that Piezo1 plays in a wide range of immune cells and its association with a variety of diseases, the search for substrates that bind specifically to Piezo1 could certainly bring about a dramatic change in the design of the corresponding drugs. However, under physiological conditions, the activity of Piezo1 is regulated only by force and there are few natural substrates. Therefore, identifying specific blockers or activators is difficult. Early studies found that gadolinium ions and ruthenium red could act as nonspecific cation channel blockers and inhibit various mechanosensitive ion channels, including Piezo channels [143]. GsMTx4 is the only specific blocker of the mechanically sensitive ion channels [144]. Six lysine residues stabilize GsMTx4 on the surface at rest; when membrane tension increases, the lateral pressure in the phospholipid bilayer decreases and the permeation distance of GsMTx4 in the cell membrane deepens, leading to a localized relaxation of the outer phospholipid molecule [145]. Patapoutian and Bandell (2015) identified a specific activator of the Piezo1 and Piezo2 channels (Yoda1) from approximately 3.25 million small molecules using a high-throughput screening method [146]. The subsequent discovery of structural analogs of Yoda1, such as Dooku1, also significantly affected Piezo1 [147]. Xiao and He (2018) screened two new Piezo channel activators, Jedi1 and Jedi2, from approximately 3000 small-molecule activators of Ca^2+^ using a high-throughput screening method [33]. King et al. (2019) showed that shear stress triggers apoptosis through Piezo1 channels via tumor necrosis factor-related apoptosis-inducing ligand. TRAIL induces explicit apoptosis of cancer cells and, combined with Yodal, leads to calcium inflow, activating calpain, which causes mitochondrial outer membrane permeabilization (MOMP) by enhancing Bax activation and ultimately sensitizing cancer cells to TRAIL [148]. However, King et al. also noted that Yoda1 may be more effective at inducing apoptosis in some metabolically active tissues (e.g., human endothelial cells) than in several types of cancer cells and that this could be improved through chemical modification and improved targeting in the future [148]. Yin et al. found that after activating Piezo1 on vascular smooth muscle cells using Yoda1, apoptosis of vascular smooth muscle cells was induced by Ca^2+^ overload, massive ROS production, and mitochondrial dysfunction [149]. Therefore, Piezo1 activators or antagonists may be used as adjuvants to enhance the efficacy of anticancer drugs. It should also be possible to improve cardiac function by controlling the fibrosis of CFs through the pharmacological inhibition of Piezo1, providing a new therapeutic direction for HF.

### 4.2. Ultrasonic Genetic Remote Modulation Switch

Ultrasound genetics technology combines the ability of the non-invasive remote control of ultrasound with the precise localization of genetics, which can achieve the non-invasive precise localization of lesions or neural sites for regulation. Ultrasound technology has been widely used in biological imaging and therapy because of its high spatial resolution and good penetration [150]. Therefore, the use of ultrasound to activate specific signaling pathways in vivo, namely ultrasound genetics, is of great interest for the development of a new generation of molecular tools.

Ultrasound can be used as a mechanical stimulus to modulate mechanosensitive ion channel switching [151,152]. Several mechanosensitive channels are activated by ultrasound at the cellular and animal levels, such as the mechanosensitive channel of large conductance (Mscl), the TRP4 channel, and the two-pore-domain potassium family (K2P) channel [153,154,155]. Current studies on the ultrasound activation of Piezo1 have focused on the in vitro cellular level, especially with regards to immune cells, tumor cells, and stem cells. Pan et al. (2017) first determined that Piezo1 could be activated by ultrasound stimulation and combined it with transcriptional gene regulation. Ultrasound activates the expression of chimeric antigen receptors in Jurkat T-cell lines and primary T cells in real- time, ultimately identifying and eradicating target tumor cells [156]. Excessive cardiac fibrosis is essential in the progression of various forms of heart disease and HF. Haig Aghajanian et al. found that T cells expressing chimeric antigen receptors (CARs) targeting FAP significantly reduced cardiac fibrosis due to hypertension or ischemic injury [157]. The generation of transient anti-fibrotic chimeric antigen receptor (CAR) T cells in vivo effectively treated cardiac injury through real-time activation of Piezo1 channels to modulate CAR-T activation to control the cardiac fibrosis process [158]. Alternatively, ultrasound can open Piezo1 channels on tumor cells, leading to Ca^2+^ entry, activating calpain activation to disrupt microtubules, and ultimately killing cancer cells [159]. Zhang et al. performed ultrasound stimulation of human umbilical vein endothelial cells (HUV) with a stimulation power of 0.2 W and a frequency of 1 MHz. They confirmed that Piezo1 might be activated by the shear stress generated by ultrasound stimulation activation. It was shown that ultrasonic stimulation could regulate the permeability of the cell membrane of HUVECs through Piezo1 [160]. Low-intensity pulsed ultrasound (LIPUS) activates increased Piezo1 expression on dental pulp stem cells (DPSCs) and promotes cell proliferation [161]. In addition, ultrasound intensity can enhance mechanical sensitivity by combining Piezo1 with sensitizers, such as microbubbles and magnetic nanobubbles, thereby reducing ultrasound intensity and cell damage [162,163]. Ultrasound activation lacks specificity for Piezo1 channels, and there may be other unknown mechanosensitive ion channels that can be activated by high-intensity ultrasound for short periods [151,164,165]. Ultrasonic modulation of Piezo1 is characterized by being non-invasive, convenient for in vitro modulation, spatially and temporally controllable, and precise. However, this technology is still in its infancy and research is focused on primary cells and cell lines cultured in vitro. Due to the existence of several mechanosensitive ion channels in vivo, it is difficult for the ultrasound to selectively modulate piezo1. In addition, ultrasonic methods for opening piezo1 in vivo are required. In vivo studies of the combination of ultrasound and Piezo1 channels remain to be enhanced, ultimately providing more rationale for their combined use in treating diseases such as HF.

## 5. Conclusions

Heart failure is the ultimate consequence of many cardiovascular diseases [166]. There are pharmacological and device-based treatments. However, they have shortcomings that may lead to failure in preserving the ejection fraction [167]. Therefore, new therapeutic options are of interest. The inadequately understood complex pathophysiological mechanisms established in the HF process and multiple hypotheses still need to yield viable targets for therapeutic action or provide effective treatments. This review proposes that Piezo1 plays an essential role in heart failure. Activation of Piezo1 promotes the differentiation of cardiac fibroblasts into myofibroblast cells and accelerates myocardial fibers [64,65,66]. Piezo1 activation leads to the release of ROS from cardiac myocytes, and pressure overload upregulates Piezo1 expression in cardiac myocytes, ultimately leading to dilated cardiomyopathy [22,71]. Activation of Piezo1 in vascular endothelial cells leads to the opening of the endothelial barrier, resulting in the infiltration of immune cells [78]. Another important factor in heart failure is the inflammatory factor burst. Piezo1 can induce the differentiation of macrophages into M1-like macrophages that release pro-inflammatory factors [103]. In addition, activation of Piezo1 on DCs leads to their secretion of pro-inflammatory factors [119]. Piezo1 activation regulates T cell differentiation and affects Treg/Th17 cell homeostasis [138]. Therefore, inhibiting Piezo1 activity to delay the progression of heart failure may be a new direction for treating heart failure. However, the mechanisms of Piezo1 in heart-failure-related diseases are still poorly studied and mainly focused on the cellular level. Applying Piezo1 activators or antagonists in animal models of heart failure remains to be explored. The future development of targeted Piezo1 drugs or combined ultrasound therapy also deserves further exploration.

## Data Availability

Not applicable.
